# Diagnostic accuracy of the aspartate aminotransferase-to-platelet ratio index for the prediction of hepatitis B-related fibrosis: a leading meta-analysis

**DOI:** 10.1186/1471-230X-12-14

**Published:** 2012-02-14

**Authors:** Wenwen Jin, Zhonghua Lin, Yongning Xin, Xiangjun Jiang, Quanjiang Dong, Shiying Xuan

**Affiliations:** 1 Qingdao Municipal Hospital, Qingdao 266021, Shandong Province, China; 2 College of Medicine and Pharmaceutics, Ocean University of China, Qingdao 266003, Shandong Province, China; 3 Qingdao City Key Laboratory of Digestive Diseases, Qingdao 266021, China

**Keywords:** APRI, HBV, liver fibrosis, diagnostic accuracy, meta-analysis

## Abstract

**Background:**

The aspartate aminotransferase-to-platelet ratio index (APRI), a tool with limited expense and widespread availability, is a promising noninvasive alternative to liver biopsy for detecting hepatic fibrosis. The objective of this study was to systematically review the performance of the APRI in predicting significant fibrosis and cirrhosis in hepatitis B-related fibrosis.

**Methods:**

Areas under summary receiver operating characteristic curves (AUROC), sensitivity and specificity were used to examine the accuracy of the APRI for the diagnosis of hepatitis B-related significant fibrosis and cirrhosis. Heterogeneity was explored using meta-regression.

**Results:**

Nine studies were included in this meta-analysis (n = 1,798). Prevalence of significant fibrosis and cirrhosis were 53.1% and 13.5%, respectively. The summary AUCs of the APRI for significant fibrosis and cirrhosis were 0.79 and 0.75, respectively. For significant fibrosis, an APRI threshold of 0.5 was 84% sensitive and 41% specific. At the cutoff of 1.5, the summary sensitivity and specificity were 49% and 84%, respectively. For cirrhosis, an APRI threshold of 1.0-1.5 was 54% sensitive and 78% specific. At the cutoff of 2.0, the summary sensitivity and specificity were 28% and 87%, respectively. Meta-regression analysis indicated that the APRI accuracy for both significant fibrosis and cirrhosis was affected by histological classification systems, but not influenced by the interval between Biopsy & APRI or blind biopsy.

**Conclusion:**

Our meta-analysis suggests that APRI show limited value in identifying hepatitis B-related significant fibrosis and cirrhosis.

## Background

Chronic hepatitis B virus (HBV), with which more than 400 million people are infected over the world, causes a worldwide health problem. It is the most common cause of acute and chronic liver disease worldwide, eventually progressing from fibrosis to cirrhosis and/or hepatocellular carcinoma [1]. It is well known that the exact staging of liver fibrosis is crucial for the therapeutic decision and assessing of the prognosis of CHB patients. Currently, liver biopsy, the gold standard, is limited by invasiveness, complications, sampling error, variability in pathological interpretation, and the reluctance of patients to undergo repeated biopsies to monitor disease progression [2,3], and 0.2-2% morbidity rate [4,5]. Because of these limitations, noninvasive measures have been examined by numerous studies to grade liver fibrosis. The aspartate aminotransferase-to-platelet ratio index (APRI) was first reported and used to identify patients with HCV-related hepatic fibrosis by Wai and his colleague in 2003 [6]. This index has the advantage of including only 2 inexpensive laboratory tests, which are performed routinely in all patients. The APRI has shown great value in predicting HCV-related fibrosis. So far, there have been several researches conducted to assess the APRI for predicting the fibrosis stage of HBV patients, and some of the existing researches are controversial. The objective of this study was to systematically review the diagnostic accuracy of the APRI for the prediction of significant fibrosis and cirrhosis in hepatitis B-related fibrosis.

## Methods

### Search Strategy

The objective of our search was to identify published manuscripts of studies examining the APRI for the prediction of HBV-related fibrosis. An electronic search, without language limitations, was completed on MEDLINE, EMBASE, and China National Knowledge Infrastructure (CNKI) including the following terms: APRI, AST, platelet, hepatitis B, AST-to-platelet ratio index, and fibrosis markers (01/2003-03/2011). Additional studies were identified via a manual search of the reference lists of relevant studies. Studies were selected if they met the following inclusion criteria:

(1) The study evaluated the performance of the APRI for the prediction of fibrosis in HBV-infected patients. Studies including patients with other causes of liver disease were included if data for HBV-infected patients could be extracted.

(2) Liver biopsy was used as the reference standard for assessing fibrosis. According to METAVIR or comparable systems, they classified the fibrosis stages F ≥ 2, F ≥ 4; the Ishak system was F ≥ 3, F ≥ 5.

(3) In order to calculate the indexes (sensitivity, specificity, positive predictive value and negative predictive value) of each cut-off point, data could be extracted to allow the construction of at least one 2 × 2 table of test performance.

(4) The study included more than 40 patients. Smaller studies were excluded because of poor reliability.

### Data Abstraction

Two reviewers (JIN and LIN) independently evaluated the study eligibility, graded quality, and extracted outcome data. Disagreements were resolved by consensus. To assess the methodological quality of the studies included in the meta-analysis, the Quality Assessment of Diagnostic Accuracy Studies score was used [7,8].

The primary outcome was the identification of significant fibrosis, defined as METAVIR [9], Batts and Ludwig [10], or Scheuer [11] stages F2 through F4 or Ishak stages F3 through F6 [12]. We also examined the identification of cirrhosis (METAVIR, Batts and Ludwig, or Scheuer F4, or Ishak F5-6).

### Data Synthesis and Analysis

For each test threshold and outcome, we tabulated the data in 2 × 2 tables to count the sensitivity and specificity. In view of previous study's primary thresholds of 0.5 and1.5 for significant fibrosis and 1.0-1.5 and 2.0 for cirrhosis, we examined the areas under summary receiver operating characteristic (SROC) curves, the summary sensitivities and specificities to provide the summary measures of test performance across all tests and these thresholds. We also calculated Q^·^(which is defined by the point where sensitivity and specificity is equal, and is the best tool to reflect the diagnosis value)to assess the diagnostic accuracy and used Random effects meta-regression [13] to examine the impact of the following factors on identifying significant fibrosis: (a) interval between Biopsy & APRI(≤ 1 week or other); (b) histopathological classification systems (METAVIR, or Scheuer, Ishak, Batts and Ludwig); (c) blind biopsy (yes vs. no). All the data were analyzed in the Meta-Disc software (version 1.4).

## Results

**Figure 1:**
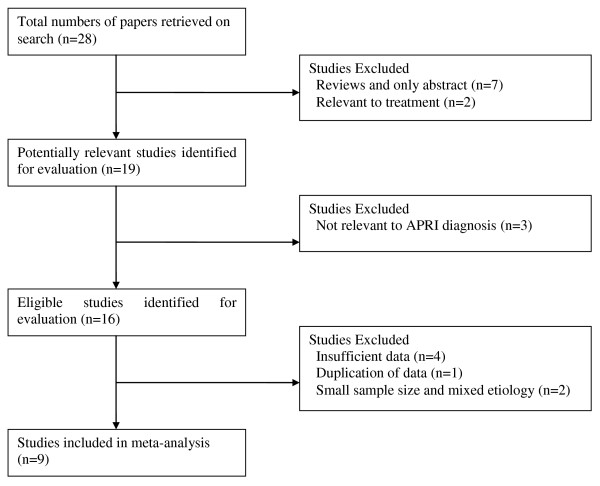
**Flow chart of article selection**.

Sixteen studies were identified that described the APRI in patients with chronic hepatitis B (Figure 1). Ultimately, 7 studies were excluded for insufficient data (n = 4) [14-17], duplication of data (n = 1) [18], small sample size or any other cause of chronic liver disease (n = 2) [19,20]. Thus, our final data set for the meta- analysis included 9 studies (Table 1) [21-29].

**Table 1:** Characteristics of the Included Studies

Author, Year, Country	Study/center Description	N	Interval Between Biopsy & APRI	Median/Mean Age, (% male)	Etiology	Liver Biopsy Description	Prevalence Significant Fibrosis (Cirrhosis)	QUADAS Score
Chrysanthosa,2006, Greece [31]	Retrospective 2 centers	205	same day	51 ± 13(74.6%)	HBV	≥ 1.5 cm	42.33%(23.11%)	14
Liu Hongbo,2007, China [33]	Retrospective 5 centers	444	≤ 1 week	Training group30(71%) Validation group28(79%)	HBV	≥ 1.0 cm	30.18%(4.50%)	14
Giada Sebastiani, 2007, Italy [10]	Retrospective 2 centers	110	same day	42.6 ± 11.3(72.7%)	HBV	≥ 1.5 cm	68.20%(20%)	14
Beom Kyung Kim 2007, Korea [35]	Retrospective 6 centers	346	same time	34 ± 14(85.3%)	HBV	4 mm	75.4%(22.8%)	14
W.G. Shin,2008, Korea [32]	Retrospective 2 centers	264	same time	24(87.1%)	HBV	17 mm	53.4%(3.4%)	14
ChenSheng, Lin 2008, China [34]	Retrospective 3 centers	48	Unclear	60.4 ± 11.3(74.2%)	HBV	Unclear	21.70%(40.20%)	12
Ruidan Zheng, 2008, China [36]	Retrospective 3 centers	131	≤ 1 week	34(84.333%)	HBV	≥ 1.0 cm	55.126%(8.897%)	14
Zhongsheng jiang 2008, China [37]	Retrospective 1 center	172	≤ 1 day	35(77.3%)	HBV	≥ 1.5 cm	83%(15%)	14
Sheng-Di Wu, 2010, China [7]	Retrospective 1 center	78	Unclear	32.6 ± 12.3 (84.6%)	HBV	≥ 15 mm	41%(11.5%)	13

### Characteristics of the Included Studies

Table 1 shows the main features of our eligible studies. A total of 1,798 patients (median age, 34.6 years; 79.4% male) were included. The overall prevalence of significant fibrosis and cirrhosis were 53.1% (range, 21.7%-83%) and 13.5% (3.4%-40.2%), respectively. Regarding histological classification systems, 5 studies used METAVIR score, 2 used Scheuer score, 1 used Ishak score, and 1 used Batts and Ludwig score. According to the Quality Assessment of Diagnostic Accuracy Studies scale, we can see that seven studies met all 14 requirements of this scale, 1 study met 13, and 1 study met 12. The methodological quality of the included studies was very good.

According to the meta-regression, the APRI accuracies for detecting significant fibrosis and cirrhosis were not affected by the interval between Biopsy & APRI (P = 0.22, P = 0.42), or blind biopsy (P = 0.09, P = 0.57), and were both influenced by histological classification systems (P = 0.01, P = 0.03),

#### Diagnostic Accuracy of the APRI for the Prediction of Significant Fibrosis

**Figure 2:**
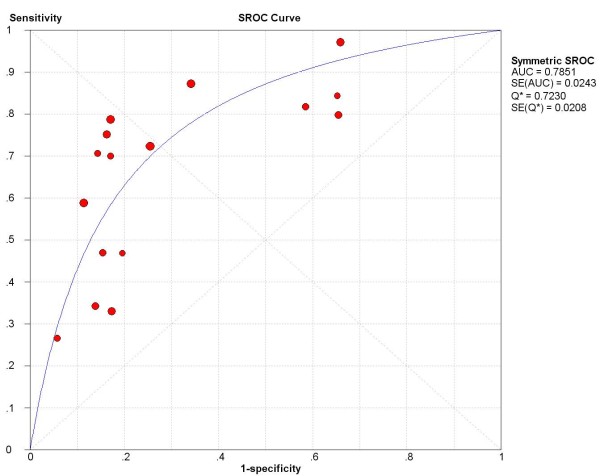
**SROC curve of the APRI for significant fibrosis**. AUC, area under the SROC curve.

Seven studies in 1,404 patients assessed the APRI for the prediction of significant fibrosis. The average prevalence of significant fibrosis in these studies was 53.1% (range, 21.7%-83%). For this outcome, the area under the SROC curve was 0.79 (SE = 0.0243) and the Q^· ^index was 0.72 (SE = 0.0208) (Figure 2). The summary sensitivities and specificities of the APRI at various thresholds for the identification of significant fibrosis were listed in Table 2. At the threshold of 0.5, the summary sensitivity and specificity were 84% (95% CI, 81%-88%) and 41% (36%-46%), respectively. At the cutoff of 1.5, the summary sensitivity and specificity were 49% (95% CI, 44%-53%) and 84% (80%-88%), respectively. Based on these data, and assuming a 53.1% prevalence of significant fibrosis (as observed in the included studies), the estimated PPV and NPV of 0.5 were 64% and 68%. At the 1.5 cutoff, the estimated PPV and NPV were 80% and 57%, respectively.

**Table 2:** Summary Sensitivities and Specificities of the APRI at various Diagnostic Thresholds for Prediction of Significant Fibrosis and Cirrhosis

Test Threshold and Outcome	Number of Studies (Patients)	Summary Sensitivity (95% CI)	Summary Specificity (95% CI)
Significant Fibrosis			
0.5	5(788)	84%(81%-88%)	41%(36%-46%)
1.5	5(788)	49%(44%-53%)	84%(80%-88%)
Cirrhosis			
1-1.5	6(1248)	54%(48%-60%)	78%(75%-80%)
2	4(792)	28%(21%-35%)	87%(84%-89%)

#### Diagnostic Accuracy of the APRI for the Prediction of Cirrhosis

**Figure 3:**
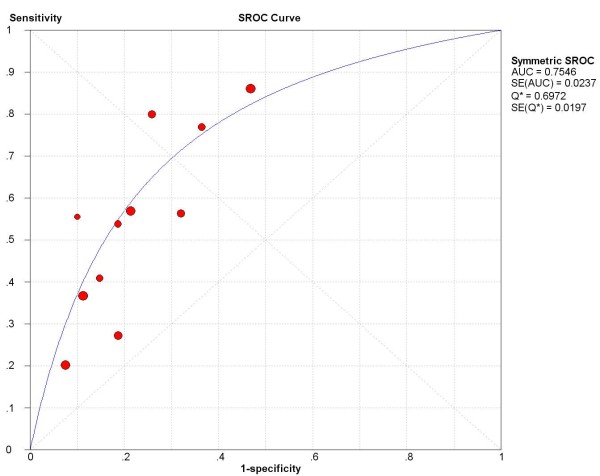
**SROC curve of the APRI for cirrhosis**. AUC, area under the SROC curve.

Six studies in 1,012 patients assessed the APRI for the prediction of cirrhosis. The average prevalence of cirrhosis in these studies was 13.5% (range, 3.4%-40.2%). For this outcome, the area under the SROC curve was 0.75 (SE = 0.0237) and the Q^· ^index was 0.70 (SE = 0.0197) (Figure 3). At the threshold of 1.0-1.5, the summary sensitivity and specificity were 54% (95% CI, 48%-60%) and 78% (75%-80%), respectively. At the cutoff of 2.0, the summary sensitivity and specificity were 28% (95% CI, 21%-35%) and 87% (84%-89%), respectively. Based on these data, and assuming a 13.5% prevalence of cirrhosis (as observed in the included studies), the estimated PPV and NPV of 1.0-1.5 were 39% and 86%, respectively. At the 2.0 cutoff, the estimated PPV and NPV were 36% and 82%, respectively.

**Figure 4:**
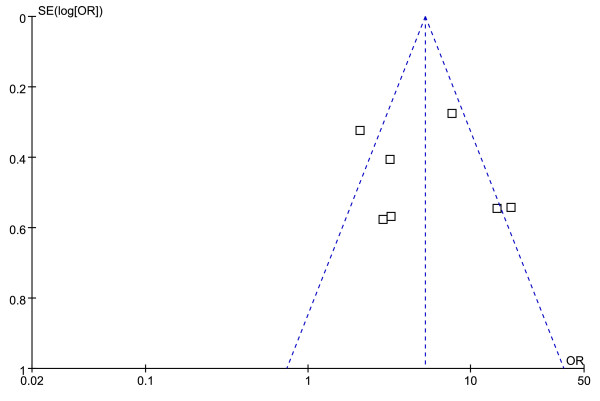
**Funnel plot of APRI in significant fibrosis to explore publication bias**.

**Figure 5:**
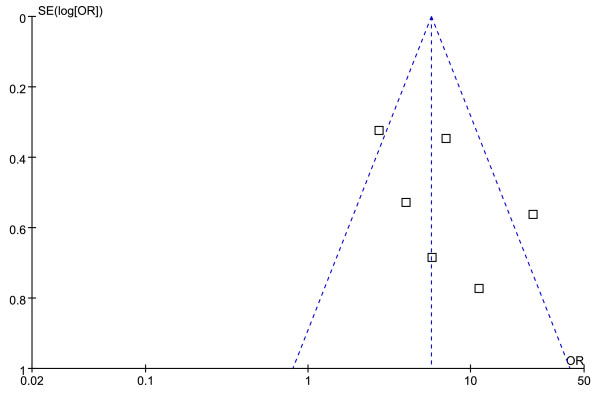
**Funnel plot of APRI in cirrhosis to explore publication bias**.

Figures 4 and 5 show the funnel plot analysis to detect the publication bias of each study for significant fibrosis and cirrhosis, respectively. The shape of the funnel plot seems to be asymmetrical, suggesting that publication bias might affect the findings of our meta-analysis.

## Discussion

Recently, the combination of FibroTest with FibroScan has demonstrated high diagnostic accuracy for the detection of significant fibrosis and cirrhosis [30]. However, the combination requires a complex instrument and is costly. Instead, the APRI is based on routinely performed, inexpensive laboratory parameters; it is potentially the ideal tool. The APRI has been derived and validated in HCV. In Wai and colleagues' original study [6], the AUC for HCV-related significant fibrosis and cirrhosis in the training and validation cohorts were 0.80 to 0.88 and 0.89 to 0.94, respectively. Subsequently, many researches supported this view [31-35]. A meta analysis researched by Abdel Aziz M et al. pointed out, in patients with chronic viral hepatitis C (CHC), the summary AUCs of the APRI for significant fibrosis and cirrhosis were 0.76 [95% CI: 0.74-0.79] and 0.82 [95%CI: 0.79-0.86], respectively; an APRI threshold of 0.5 was 81% sensitive and 50% specific, at a 40% prevalence of significant fibrosis, this threshold had a negative predictive value (NPV) of 80%. For cirrhosis, a threshold of 1.0 was 76% sensitive and 71% specific, at a 15% cirrhosis prevalence, the NPV of this threshold was 91%, the major strength of the APRI is the exclusion of significant HCV-related fibrosis [36].

In contrast to HCV, Wai et al. [14] evaluated the accuracy of models (ALT, AST and APRI) from HCV in 218 HBV patients, which indicated that non-invasive markers in predicting histology from CHC patients were unsuitable for CHB patients. Subsequent research demonstrated that in contrast to APRI, ASPRI was accurate in predicting cirrhosis and has the potential to reduce the number of liver biopsies in CHB patients when screening with ASPRI [27]. However, W.G. Shin and colleagues' study indicated that of indirect markers, the APRI yielded the best area (0.86) under the receiver operating characteristic curve [95% CI: 0.82-0.91], the APRI may be the most accurate and simple marker for predicting significant fibrosis in chronic hepatitis B [24]. Lin CS and colleagues' research supported this view [26]. Thus, the present study conclusions are controversial. In our systematic review, we summarize the diagnostic accuracy of the APRI for the prediction of HBV-related fibrosis. In our systematic review, we calculated summary sensitivities and specificities at various thresholds to translate all point estimates into clinical practice. The summary AUROC of the APRI for the diagnosis of significant fibrosis was 0.79. Moreover, the 0.5 threshold was 84% sensitive and 41% specific. Assuming a 53.1% prevalence of significant fibrosis, this translates into estimated PPV and NPV of 64% and 68%, respectively. On the contrary, a cutoff of 1.5 was less sensitive (49%) and more specific (84%). Assuming a 53.1% prevalence of significant fibrosis, this translates into estimated 80%PPV and 57% NPV. With regard to cirrhosis, the summary AUROC was 0.75. Moreover, the1.0-1.5 threshold was 54% sensitive and 78% specific. Assuming a 13.5% prevalence of cirrhosis, this translates into estimated PPV and NPV of 39% and 86%, respectively. On the contrary, a cutoff of 2.0 was less sensitive (28%) and nearly specific (87%). Assuming a 13.5% prevalence of cirrhosis, this translates into estimated 36% PPV and 82% NPV. A diagnostic tool is considered as good if the AUROC is greater than 80%, excellent if the AUROC is greater than 90% and perfect if the AUROC is 100%. According to these results, the APRI may not be a good tool for predicting significant fibrosis and cirrhosis in hepatitis B-related fibrosis and can not reduce the number of liver biopsy.

A strength of our review is that meta-regression analyses have been used for exploring factors that may be responsible for heterogeneity. We analyzed carefully three indicators that might contribute to heterogeneity: (a) the interval between Biopsy & APRI (≤ 1 week or other); (b) blind biopsy (yes VS. no); (c) histological classification systems (METAVIR, Scheuer, Ishak, Batts and Ludwig). By meta-regression, we could see that for both significant fibrosis and cirrhosis, histological classification systems were found to provide heterogeneity to summary test results. Previous research has shown that the hypothesis of the liver biopsy is 80% - 90% accurate, the AUC of medical tests cannot reach 0.9, and more likely fluctuated from 0.75 to 0.9 [37]. To solve the problem, one of the ways is improving the quality of liver biopsies.

Our study has several limitations. Firstly, only HBV-infected patients have been analyzed. Losing some patients with mix infections (HBV/HCV, HBV/HIV and HBV/NAFLD) suggest reduced accuracy. Secondly, some of the studies reported APRI thresholds not included in the original description (Table 2). For example, W.G.Shin et al. proposed that the 1.4 cutoff appears promising (79% sensitive; 83% specific for significant fibrosis) [24]. The number of studies is so small that we have not focused on these thresholds. Finally, we have chosen inclusions only by published manuscripts, so bias in the selection of search channels may influence the results to some extent.

In summary, our systematic review suggests that APRI show limited value in identifying hepatitis B-related significant fibrosis and cirrhosis. The APRI is not an appropriate choice for HBV patients to identify hepatitis B-related fibrosis in regions with limited health care resources. Future studies are necessary to identify high accuracy, cost-effectiveness and widely available measures.

## Conclusion

Our meta-analysis suggests that APRI show limited value in identifying hepatitis B-related significant fibrosis and cirrhosis.

## Competing interests

The authors declare that they have no competing interests.

## Authors' contributions

WWJ and ZHL carried out the design of this meta-analysis, conducted the searching, extracted data, analyzed the data and drafted the manuscript. YNX and SYX participated in study design and the critical revision of the manuscript. XJJ and QJD participated in the critical revision of the manuscript. All authors read and approved the final manuscript.

## Pre-publication history

The pre-publication history for this paper can be accessed here:

http://www.biomedcentral.com/1471-230X/12/14/prepub
